# Modified Brostrom-Gould surgical procedure for chronic lateral ankle instability compared with other operations: a systematic review and meta-analysis

**DOI:** 10.1186/s12891-022-05957-8

**Published:** 2022-11-12

**Authors:** Qifan Yang, Jing Liu, Chengyan Liu, Pengcheng Zhou, Dong Zhu

**Affiliations:** 1 Department of Orthopedics, the First Hospital of Jilin University, Street Xinmin 71, Changchun, China; 2 The First Clinical Medical College of Bin Zhou Medical College, Street Huanghe 661, Yantai, China

**Keywords:** Chronic lateral ankle instability, MBG, CLAI, Meta-analysis

## Abstract

**Background:**

This study performed a randomized trial data meta-analysis to assess The Modified Brostrom-Gould (MBG) for proven chronic lateral ankle instability (CLAI).

**Methods:**

All published randomized clinical trials comparing MBG and other operations were found by searching the Cochrane Library, EMBASE, and PubMed databases. The Review Manager 5.4 software was used to compare the two groups regarding postoperative functional score, ankle stability, and complications. Risk Ratio (RR) and Mean Differences (MD) were used in meta-analyses.

**Results:**

8 experiments are suitable for it, 426 patients were enrolled, and 222 patients underwent other operations surgery. Among the six outcome indicators, in terms of FAOS scores, the other operations group has an advantage, 6.53 points higher than MBG; others show no significant differences.

**Conclusions:**

Based on this meta-analysis, the authors believe that other surgical groups can achieve better outcomes than MBG in some aspects of CLAI treatment.

## Introduction

Ankle sprains are one of the most prevalent sports injuries, accounting for 15% to 20% of all injuries [1]. While most patients can return to their everyday lives after nonsurgical therapy, some persons suffer CLAI. Patients with sprained ankles often undergo extensive rehabilitation training before being diagnosed. However, more than half of the patients will eventually develop CLAI, leading to talar cartilage injury and early ankle osteoarthritis [2].

The repair of ligaments in postoperative patients depends on the tissues that have been injured; Such conditions require surgical treatment to improve clinical symptoms, improve the ability of daily living, and improve prognosis. MBG is a surgical method for treating CLAI and has long been the first-line choice. However, in recent years, for the treatment of this type of patient, some new surgical methods have emerged, such as LARS, Suture-tape augmentation, Arthroscopic suture-tape internal bracing, the Chrisman-Snook procedure, etc. [3–7]. Many scholars have compared these surgical methods with MBG and conducted RCTs for many years. For example, in 2015 and 2019, Porter M compared the two surgical methods of LARS and MBG through randomized controlled trials. Through up to 2 years and 5 years of follow-up, he found that LARS performed better in FAOS and Tenger activity scores [4, 7]; Kulwin in the 2021 article, showed that through a two-year randomized controlled trial, it was found that the suture tape can reach preinjury level of activity (RTPAL) faster and have a lower chance of complications compared with MBG. In this article, we grouped them into other surgical groups to explore how these surgical modalities differed from the MBG group. The specific surgical steps are shown in Table 1. Many scholars have compared these surgical methods with MBG and conducted RCTs for many years. The trial period included in the previous meta-analysis was relatively long. There is still a lack of evidence to support whether MBG can be the first choice for the treatment of CLAI. New randomized controlled trial evidence has emerged recently, so a new meta-analysis is needed. Analyze Other operations in-depth to give evidence-based support for medical decisions.

**Table 1:** The specific surgical procedure

Surgical procedure	Surgical procedure	Advantages	Shortcoming
LARS	LARS surgery uses a single incision and approach similar to MBG, adding two 1 cm incisions. Use the most distal incision to fix the CFL limb of the LARS to the natural attachment point on the calcaneus, use the proximal incision to pull the LARS ring over the fibular tunnel, and drill a strip through the fibula where the ATFL and CFL attachments overlap. 5 mm tunnel, targeting the proximal and posterior sides of the center of the fibula. At the end of the ATFL, make a 1 cm incision. Drill a tunnel. Then, it is advanced into the fibular tunnel. At the midpoint between the tip of the lateral malleolus and the sole, a 1 cm horizontal incision is made parallel to the posterior surface of the fibula. The surgeon drilled another tunnel, and the two limbs of the LARS were anchored to their respective distal attachment points with anchors. The proximal end of the anchor is passed along the fibular tunnel, tension is applied to the two limbs separately to form a stable ankle joint, and a third anchor is used for final fixation. Finally, close the wound	1. It is beneficial to the healing of the lateral collateral ligament of the ankle joint 2. It provides higher fixation strength than MBG surgery	1. Higher incidence of foreign body is and osteoarthropathy
The surgical procedure described by Karlsson et al	The ligaments and joint capsule are separated approximately 1–2 mm from the anterior and inferior borders of the fibula. After the surgeon lifted the periosteal flap of the fibula, a small bone fragment was chiseled about 4 × 4 mm from the anterior and lateral sides of the fibula—drill holes in the fibula with a 2.0 mm drill. The ligaments and joint capsule are shortened to an appropriate length and sutured to the bone. Tighten the stitches, so the foot is in a neutral position. Finally, the surgeon performed duplication with a periosteal flap and the proximal end of the ligament to strengthen the reconstruction	1. Simple operation 2. Simple technique 3. Small economic cost 4. Fewer complications	1. High requirements for local residual ligaments
The Chrisman-Snook procedure	A posterior curved incision is made from 4 to 5 cm proximal to the top of the fibula to 2 cm proximal to the top of the fifth metatarsal. A flap is formed. The medial half of the peroneus brevis tendon was taken as a graft, retaining its ligamentous attachment to the fifth metatarsal. Use a drill to drill a bone tunnel through the fibula. The separated tendon is passed through the bone tunnel and attached to the junction of the calcaneus with the calcaneofibular ligament. With the ankle dorsiflexed 10°, the graft is tensioned and secured to the calcaneus, and the end of the graft is sutured to the anterior portion of the anterior talofibular ligament	1. Effectively limit the inclination of the talus, which is a good simulation of ankle motion similar to the intact ligament	1. Inability to fully restore the role of the pre-injured ligaments
MBG + ST	An oblique longitudinal incision was made from the posterior end of the fibula in the direction of the fourth metatarsal. The surgeon lifted the 1 cm periosteum from the distal end of the fibula to the proximal end, and the residual ATFL and CFL were cut off. Insert an anchor along the insertion point on the lateral wall of the ATFL talus and the corresponding location on the fibula. The surgeon inserted two more suture anchors, one at 5 mm proximal to the CFL and the other at 18 mm. The periosteal flap combines the inferior extensor retinaculum with absorbable sutures, and the skin is sutured	1. Provides higher fixation strength than MBG surgery, and can reach pre-injury movement levels faster 2. Low risk of complications	1. Higher cost of surgery
MBG	A curved incision was made behind the fibula to repair the damaged ligament directly, and the unique bundle of the IER was sutured to the lower segment of the fibula	1. The technology is mature and more people accept it	1. Extensive exposure, easy to damage nerves 2. It is not effective for patients with high BMI and extensive ligament laxity

This study aims to demonstrate that other operations can achieve better outcomes than MBG in treating certain CLAIs, not only insisting on the idea that MBG is the gold standard for treating CLAI. To our knowledge, this is the first meta-analysis to incorporate recent randomized controlled trials evaluating both MBG and many procedures.

## Material and method

### Study selection and search strategy

This meta-analysis was implemented by the PRISMA guidelines (the preferred reporting item for systematic reviews and meta-analysis) [8]. the ID is CRD42021248704. As of March 1, 2021, we searched various electronic databases, including the Cochrane Library, PubMed, and Embase. Each database was independently searched by two reviewers (Qifan Yang and Jing Liu) using the following research methodology: ((((((Ankle[MESH]) OR (Ankle[Title/Abstract])) OR (Lateral Ligament, Ankle[MESH])) OR (Lateral Ligament, Ankle[Title/Abstract])) OR (ankle*[Title/Abstract] AND (injur*[Title/Abstract] OR joint*[Title/Abstract] OR ligament*[Title/Abstract] OR sprain*[Title/Abstract] OR strain*[Title/Abstract] OR inversion*[Title/Abstract] OR rupture*[Title/Abstract] OR tear*[Title/Abstract] OR torn)[Title/Abstract])) AND ((Ligaments, Articular[MESH]) OR (Ligaments, Articular[Title/Abstract]))) AND (((((instability[Title/Abstract] OR unstable[Title/Abstract] OR lax*[Title/Abstract] OR recurrent[Title/Abstract] OR chronic*[Title/Abstract]) OR (Joint Instability[MESH])) OR (Joint Instability[Title/Abstract])) OR (Chronic Disease[MESH])) OR (Chronic Disease[Title/Abstract])) AND (randomized controlled trial[Filter]). The senior author will resolve any disagreements (Dong Zhu). We looked at the title and abstract of each article we found and then read the complete text of the studies that fulfilled the criteria. Also, I looked through the reference list of the included literature to see whether there was any research that met the criteria. There are no limitations on the location of study, the type of research, or the status of publications.

### Eligibility criteria

All prospective randomized controlled trials evaluating the clinical outcomes of other operations vs. MBG in the treatment of CLAI are included. (1) Randomized, double-blind, placebo-controlled trials; (2) Studies with at least one of the following clinical outcomes: Ankle Outcome Score (FAOS), Ankle Joint Stability, Complications;(3) Research in full text is available for reading. Studies with patients who have had any prior ankle surgery, studies without full text, and studies with more than 20% of patients lost to follow-up are excluded.

### Study quality assessment

Two reviewers (Qifan Yang and Jing Liu) independently evaluated the quality of identified RCTs using the Cochrane Library's risk of bias [9], which included seven items: random number generation, concealment of allocation schemes, blinding of experimenters and participants, non-conforming result data, selective reporting, and other deviations. The studies were all categorized as low, unclear, or high risk. The senior author will decide on any differences between the two reviewers.

### Data extraction

Two reviewers (Qifan Yang and Jing Liu) independently collected all relevant data from the study in a predetermined way. Data in other formats (such as mean and range) should be translated to mean ± SD using the Cochrane Handbook Standard Deviation guidelines [10]. The article data includes the first author's name, the year of publication, the study title, the sample size, and the follow-up date. The most prevalent demographic data points are average age, gender, and diagnosis. Clinical prognostic indicators include Foot and Ankle Prognosis Score (FAOS), ankle joint stability and complications. The senior author decides whether there is a disagreement between the two reviewers.

### Statistical analysis

Review Manager (Revman, Version 5.4, The Cochrane Collaboration) was used to analyze the data. For continuous data, mean difference (MD) and 95% CI intervals (95% CI) were calculated, whereas, for dichotomous data, odds ratios (ORs) and 95% CI were used. I^2^ tests were employed to determine heterogeneity, with I^2^ > 50% and *P* < 0.10 indicating considerable heterogeneity. When significant heterogeneity was identified, a random-effect model was used; otherwise, a fixed-effect model was used. Statistical significance was defined as a *P* value of less than 0.05.

## Results

### Litterateurs review

**Fig. 1:**
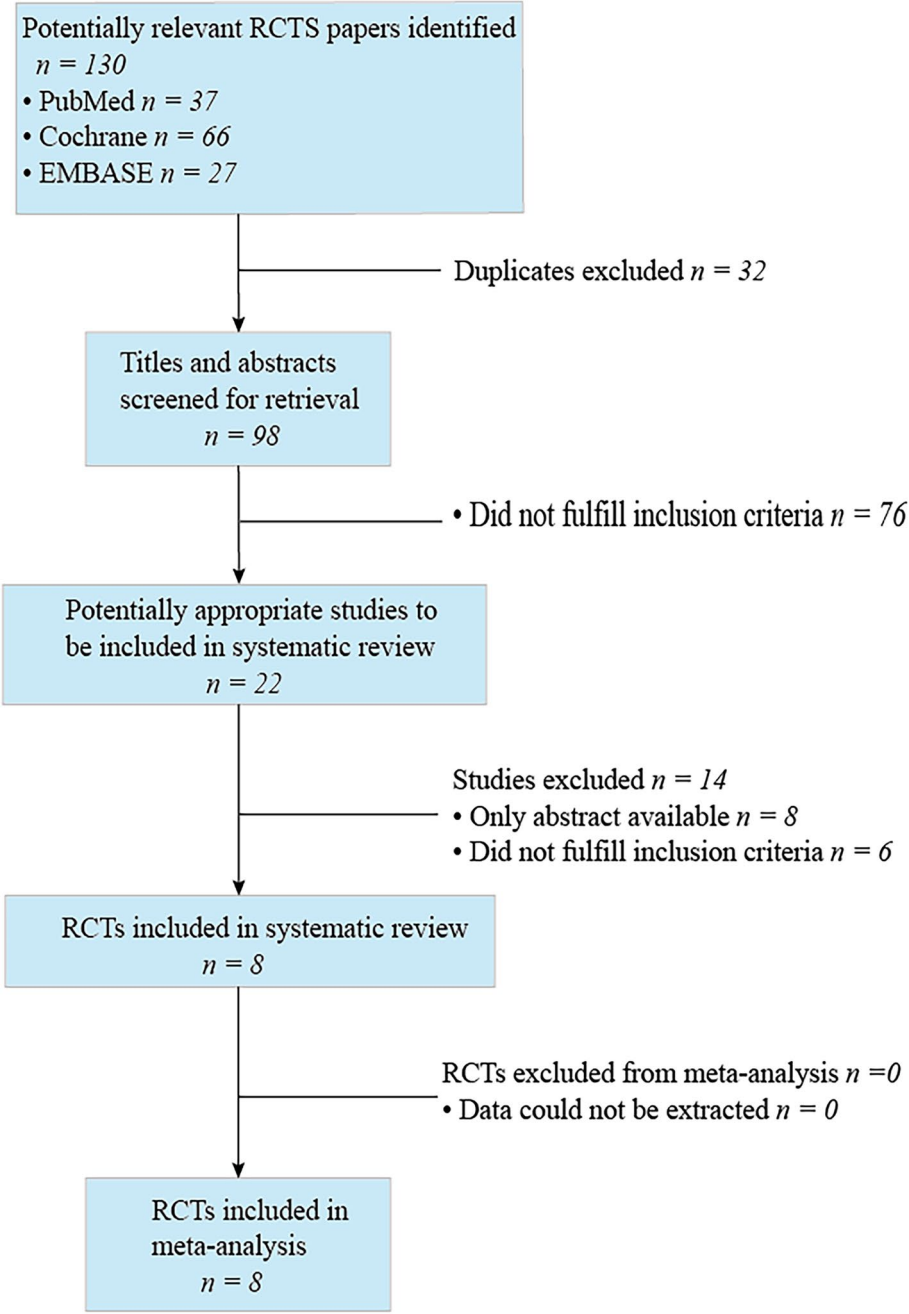
Study selection pipeline diagram for PRISMA (Preferred Reporting Items for Systemic Meta-Analyses)

After an initial search of relevant databases, 130 papers meeting the essential screening criteria were found, as shown in Fig. 1. The inclusion and exclusion criteria were applied to 22 research, and duplicate papers were deleted. Then 22 full texts were assessed for eligibility, and 8 RCTs [4, 5, 7, 10–14] with several 426 patients were included in this meta-analysis.

### Studies characterization

**Fig. 2:**
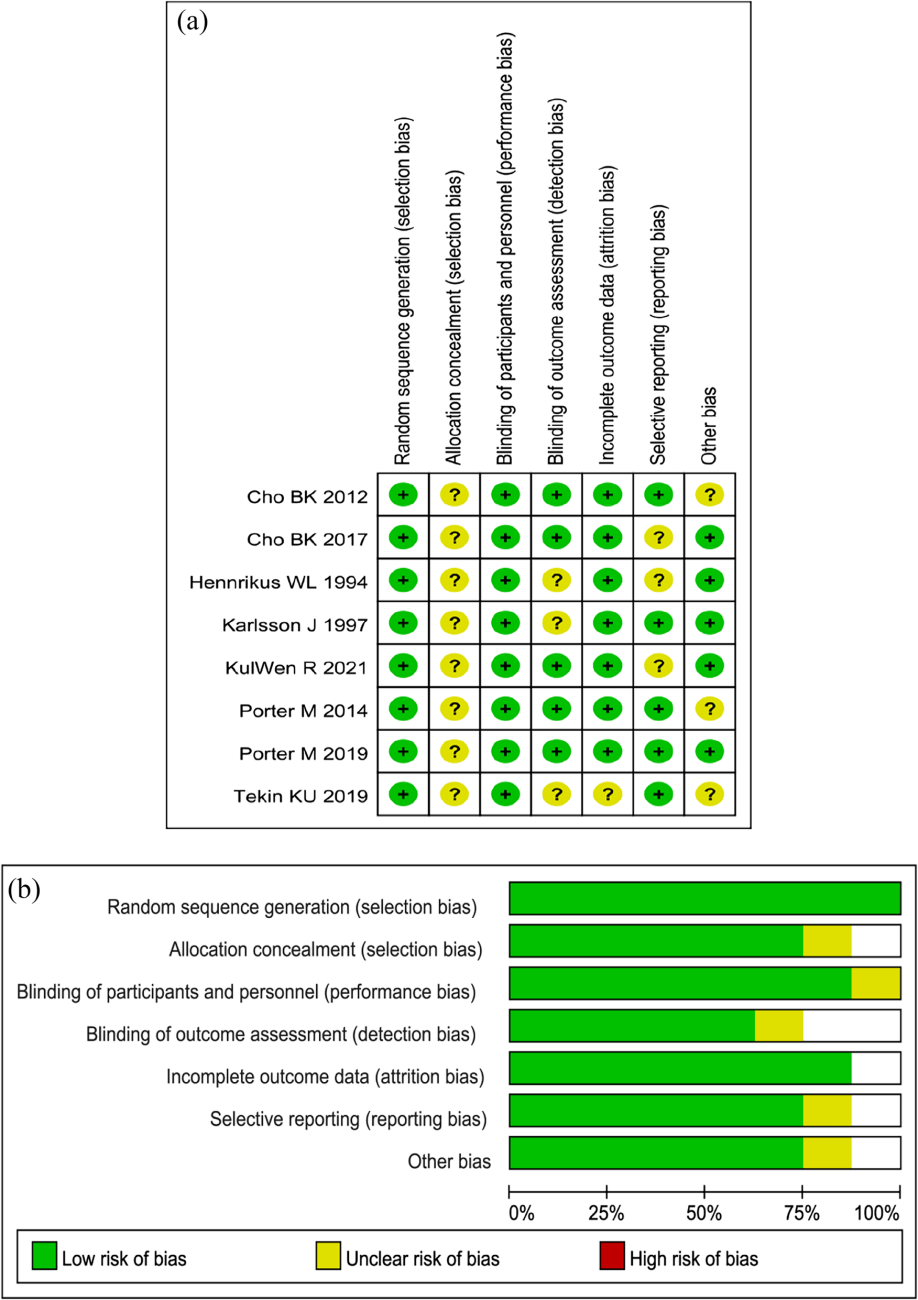
The Cochrane Library for RCTs' Risk of Bias test was used to assess the quality of the RCTs that were included. **a** Author judgments on each risk of bias item for each included study, and **b** percentages for each risk of bias item across all included studies

Table 2 illustrates the general information about the studies that were included. All of the randomized controlled trials were published between 1994 and 2021, with a one minimum follow-up time. A total of 426 patients with CLAI were included in the study, with 222 receiving other operations treatment and 204 receiving MBG treatment. The average age of the two groups was not significantly different (*P* > 0.05). Figure 2 summarizes the findings of the quality assessment.

**Table 2:** Baseline data from the studies that were included

Study	Country	Design	Other operations group			MBG group			Outcome	Measurement timepoint (month)
			Sample size	Age (year)	Female (%)	Sample size	Age (year)	Female (%)		
Ulku TK, 2020 [15]	Australia	RCT, 2 arms	22	26.1 ± 8.3	54.55%	25	24.0 ± 6.9	52.00%	FAOS Score、Tegner Score	12、24、60
Porter M, 2019 [4]	Australia	RCT, 2 arms	21	26.1	48%	20	24	50%	FAOS Score	12、24
Porter M, 2015 [7]	korea	RCT, 2 arms	28	26.6	100%	27	28.1	100%	FAOS Score、FAAM Score、Anterior Talar Translation And Talar Tilt	3、24
Kulwin R, 2021 [13]	Korea	RCT, 2 arms	20	30.7	45%	20	33.9	40%	Karlsson Score、Sefton Score、Anterior Talar Translation And Talar Tilt	3、12、24
Karlsson J, 1997 [6]	Turkey	RCT, 2 arms	30	27.8	_	31	28.6		FAOS Score、 FAAM Score	12
Hennrikus WL, 1996 [5]	Sweden	RCT, 2 arms	30	24	_	30	24		Karlsson and Peterson Score、Tegner Score、Anterior Talar Translation And Talar Tilt	36
Cho BK, 2017 [16]	California	RCT, 2 arms	20	26	0%	20	25	29%	Sefton Score、Talar Tilt	29
Cho BK, 2012 [12]	USA	RCT, 2 arms	60	31.3 ± 15.5	54%	59	41.4 ± 14.0	78%	RTPAL、VR12M、VR12P、FAAM ADL、FAAM Sports、KP、VAS	_

### Meta-analysis of outcome

#### FAOS – Pain, symptoms, activities of daily living (ADLs), sport, quality of life (QOL), and total scores

##### Pain

**Fig. 3:**
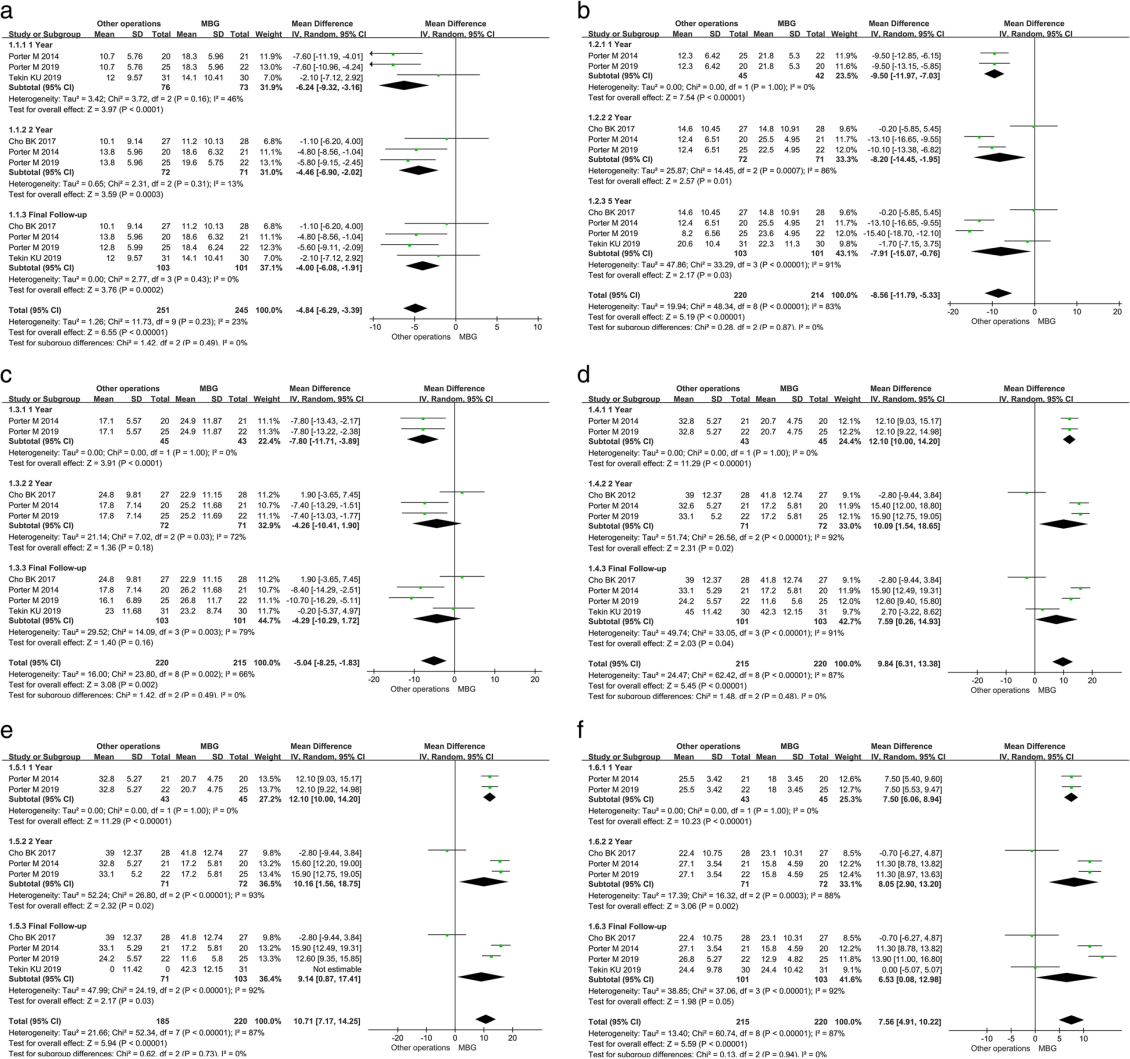
Between MBG and Other operations, a forest plot of different sections of the Foot and Ankle Outcome Score (FAOS) was created. In the elements of Pain (**a**), Symptoms (**b**), Activities of daily living (ADL) (**c**), Sports (**d**), Quality of life (QOL) (**e**), and Total scores (**f**), the forest plot demonstrates that the summary points are slanted towards other operations

The FAOS pain score with SD was studied in four trials [4, 10, 14, 17], totaling 204 participants. The results showed that there was no considerable disparity (*P* = 0.43, I^2^ = 0%). Hence the data was analyzed using the random-effects model. There was a significant difference in pain ratings between the other operations and MBG groups (MD = -4.00, 95% CI: -6.08— -1.91, *P* = 0.0002) in the summary data. The other operations group had a better clinical score influence; Then, according to follow-up time, the other operations group had a better clinical scoring effect. The data was broken down into subgroups and analyzed. First, 149 individuals in three studies [4, 14, 17]reported the FAOS pain score after being monitored for a year. Since there was no significant heterogeneity (*P* = 0.16, I^2^ = 46), the findings were analyzed using the random-effects model. The pain scores of the Other operations and MBG groups were significantly different (MD = -6.24, 95% CI: -9.32- -3.16, *P* < 0.0001); When the follow-up time was two years, three items were significantly different. The study [10, 14, 17] included 143 patients who had FAOS with SD pain scores. The findings show no significant heterogeneity (*P* = 0.31, I^2^ = 13%), and the data analysis is conducted using a random-effects model. The Other operations group outperformed the MBG group (MD = -4.46, 95% CI: -6.90- -2.02, *P* = 0.0003) (Fig. 3a). The authors believe that the difference in FAOS scores is due to the use of tendons or suture tape to mimic the torn ligament's original physiological role and enhance ankle stability with more muscular tissue or material, thereby causing the difference in FAOS results.

##### Symptoms

The symptom elements of the FAOS score were reported as MD ± SD in four studies (204 individuals) [4, 10, 14, 17]. Figure 3b depicts the results. Heterogeneity is statistically significant (*P* < 0.00001, I^2^ = 83%). The data analysis is completed using the random-effects model, and there is a distinction between table the two groups (MD = -7.91, 95%CI: -15.07- -0.76, *P* = 0.03). Subgroup analysis based on follow-up time was used to determine the FAOS symptom score and SD; two studies [4, 17] with 87 patients reported the FAOS symptom score and SD at one year. Because there was no significant heterogeneity (*P* = 1.00, I^2^ = 0%), the data were analyzed using the random-effects model. According to the summary data, there was a significant difference in symptom scores between the Other operations and MBG groups (MD = -9.50, 95%CI: -11.96- -7.03, *P* < 0.00001). After a two-year follow-up period, 143 patients were involved in three trials [4, 10, 17]. Because of the substantial heterogeneity (*P* = 0.0007, I^2^ = 86%), the data were analyzed using the random-effects model. The Other operations group had a higher score (MD = -8.20, 95% CI: -14.45- -1.95, *P* = 0.01) than the MBG group.

##### ADLs

Four articles [4, 10, 14, 17] included 204 patients and provided the results of the FAOS ADL component in the form of MD ± SD for analysis. Due to the strong heterogeneity of the results (*P* = 0.003, I^2^ = 66%), the random-effects model was used in the analysis, and the results revealed that the difference between the groups was not statistically significant (MD = -4.29, 95% CI: -10.29- 1.72, *P* = 0.18). Following that, a subgroup analysis was performed. After a year of follow-up, 88 patients in two trials [4, 17] revealed no significant heterogeneity in the ADLS score of FAOS (*P* = 1.00, I^2^ = 0%), and the data analysis was randomized Effect model. The ADLS scores of the Other operations and MBG groups were substantially different (MD = -7.60, 95%CI: -3.89- -11.71, *P* < 0.00001), according to the summary results. Three trials [4, 10, 17] involved 143 patients when the period was two years. Because the data is highly heterogeneous (*P* = 0.03, I^2^ = 72%), the random-effects model is used to examine it. According to the summary data, the difference in ADLS scores between the MBG and Other operations groups was not statistically significant (MD = -4.26, 95%CI: -10.41- 1.90, *P* = 0.18). (Fig. 3c).

##### Sport

Data from four studies [4, 10, 14, 17] are provided for the FAOS sports scores. Because of the considerable heterogeneity (*P* < 0.00001, I^2^ = 91%), we applied a random-effects model to examine the data and discovered that there was a statistical difference between the Other operations and MBG groups (MD = 7.69, 95%CI: 0.26- 14.93, *P* < 0.00001). For various periods, a subgroup assessment was conducted. A total of 88 patients in two investigations [4, 17] reported FAOS exercise scores after a year of follow-up, with no significant heterogeneity in the results (*P* = 1.00, I^2^ = 0%). The data were analyzed using a random-effects model, which indicated a significant variation in Sports ratings between the Other operations and MBG groups (MD = 12.10, 95% CI: 10.00–14.20, *P* < 0.00001). The follow-up period was two years, with three items. There were 143 patients in the study [4, 10, 17]. It has significant heterogeneity (*P* < 0.00001, I^2^ = 92%), and data analysis is done using the random-effects model. The Other operations group outperformed the MBG group in Sports scores (MD = 10.09, 95% CI: 1.54- 18.65, *P* = 0.02), according to the summary data (Fig. 3d). The authors believe that the difference in FAOS scores is due to the use of tendons or suture tape to mimic the torn ligament's original physiological role and enhance ankle stability with more muscular tissue or material, thereby causing the difference in FAOS results.

##### QOL

In four various studies, the FAOS QOL score was recorded. [4–7] Because the variability was significant (*P* < 0.00001, I^2^ = 92%), a random-effects meta-analysis was used, which can be seen in Fig. 3e. There was a significant difference between the two groups (MD = 9.14, 95% CI: 0.87–17.41, *P* = 0.03). After a one-year follow-up period, two studies [4, 17]with 88 patients reported the FAOS QOL score with SD. The data were examined using a random-effect model because there was no substantial variation (*P* = 1.00, I^2^ = 0%). There was a significant difference between the Other operations and MBG groups in terms of Sports score (MD = 12.10, 95%CI: 10.00–14.20, *P* < 0.00001). Three investigations [4, 10, 17] involving 143 patients were conducted after two years of follow-up. Because of the significant heterogeneity (*P* < 0.00001, I^2^ = 93%), the data were analyzed using a random-effect model. The difference in QOL score between the other operations and MBG groups was considerable (MD = 10.16, 95%CI: 1.56- 18.75, *P* = 0.02), according to the pooled data.

##### Total scores

Four studies were selected for data synthesis in AFOS Total Scores [4, 10, 14, 17]. We pooled the data using a random-effect model (Fig. 3f) due to significant heterogeneity (*P* < 0.00001, I^2^ = 87%) and discovered a significant difference (MD = 6.53, 95% CI: 0.08- 12.08, *P* = 0.05). After one year of follow-up, two studies [4, 17] involving 88 people revealed total FAOS scores with SD. The statistical analyses were conducted using a random-effect model (*P* = 1.00, I^2^ = 0%). Three studies [4, 10, 17] involving 143 patients found that the difference between MBG and Other operations groups was significant in terms of Total scores (MD = 7.50, 95%CI: 6.06– 8.94, *P* < 0.00001) during one year follow-up period. The data were determined using a random-effect model due to the substantial heterogeneity (*P* = 0.0003, I^2^ = 88%). The variation scores between the MBG and Other operations groups were notable (MD = 8.05, 95%CI: 2.90- 13.20, *P* = 0.002) during a two-year follow-up period.

#### Ankle stability–talar tilt angle and anterior talar translation

##### Talar tilt angle

**Fig. 4:**
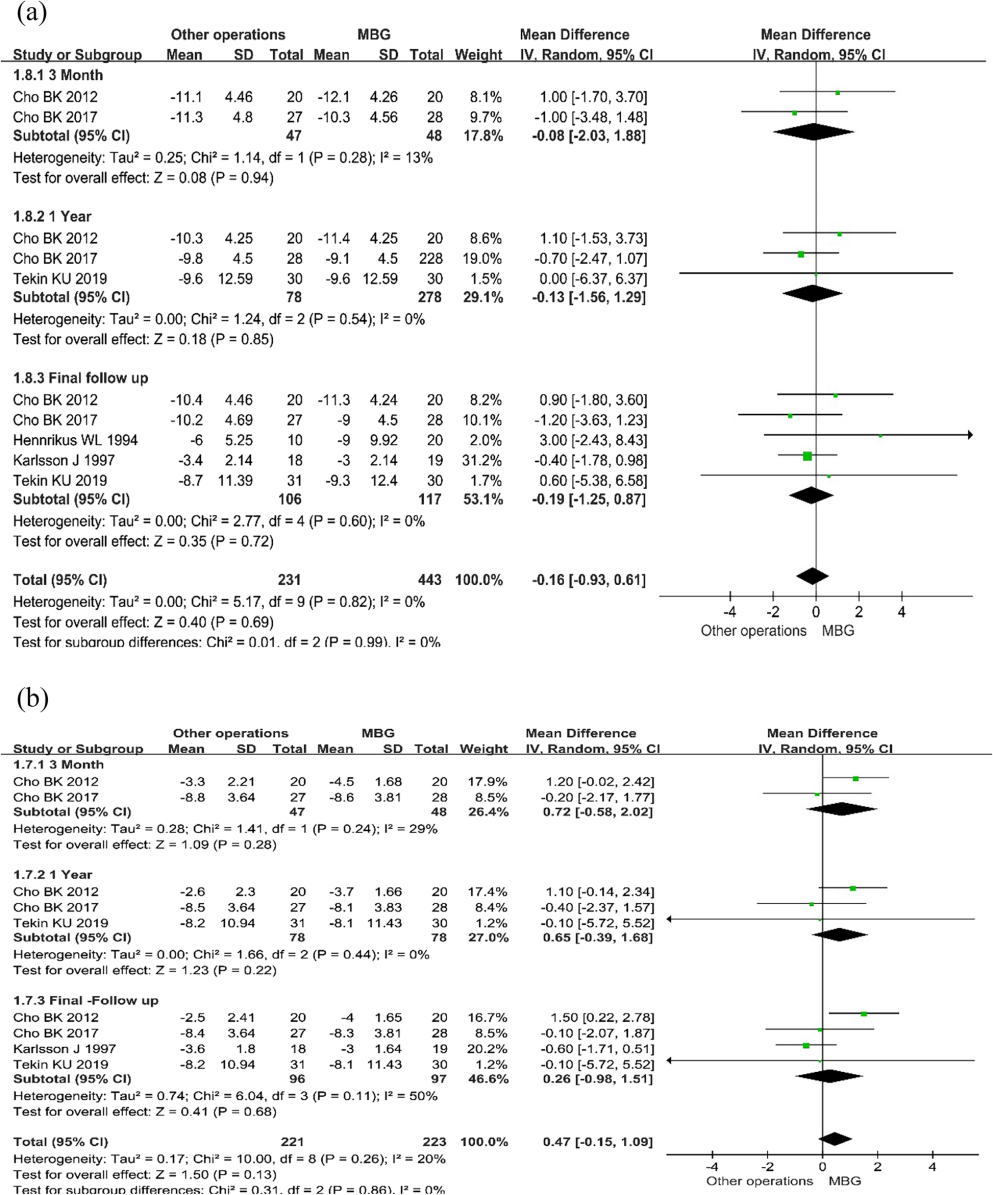
The pooled results of ankle stability evaluated with stress radiographs: Forest plot of talar tilt angle (**a**) and anterior talar translation (**b**) showing no significant difference between two groups

The Talar tilt angle was reported in five investigations [5, 6, 13, 18]. There was no significant heterogeneity (*P* = 0.82, I^2^ = 0%), and the distinction was not statistically significant (MD = -0.19, 95%CI: -1.25– 0.87, *P* = 0.72). Figure 4a shows that after a three-month follow-up term, two investigations [10, 12] involving 95 patients reported the talar tilt angle with SD. Because there was no significant variation (*P* = 0.28, I^2^ = 13%), the data were collected using a random-effect model. Three investigations [4, 10, 14] comprising 156 patients found no significant variations in Talar tilt angle between MBG and Other operations groups after a year of follow-up (MD = -0.08, 95% CI: -2.03– 1.88, *P* = 0.94). The data were analyzed using the random-effect approach, and there was no considerable disparity (*P* = 0.54, I^2^ = 0%). According to the combined results, there was no great disparity in Talar tilt angle between the MBG and Other operations groups (MD = -0.13, 95%CI: -1.56– 1.29, *P* = 0.85).

##### Anterior talar translation

Data on anterior talar translation was gathered from 193 people in four studies [6, 10, 12, 14]. Considering the lack of heterogeneity (*P* = 0.11, I^2^ = 50%), a fixed model was used to merge the data, as seen in Fig. 4b, and no statistical significance was found (MD = 0.26, 95%CI: -0.98– 1.51, *P* = 0.68). After a three month follow-up period, two studies [10, 12] involving 95 patients reported a Sports score of FAOS with SD. There was no considerable disparity (*P* = 0.24, I^2^ = 29%). In terms of anterior talar translation, there was no massive distinction between the MBG and Other operations groups (MD = 0.72, 95% CI: -0.58– 2.02, *P* = 0.28). After a year of follow-up, three investigations [4, 12, 14] with 156 participants were completed. Because there was no significant heterogeneity (*P* = 0.44, I^2^ = 0%), the data were analyzed using a random-effect model. There were no substantial distinction groups (MD = 0.65, 95%CI: -0.39–1.68, *P* = 0.22), according to the combined data.

#### Postoperative complications

**Fig. 5:**
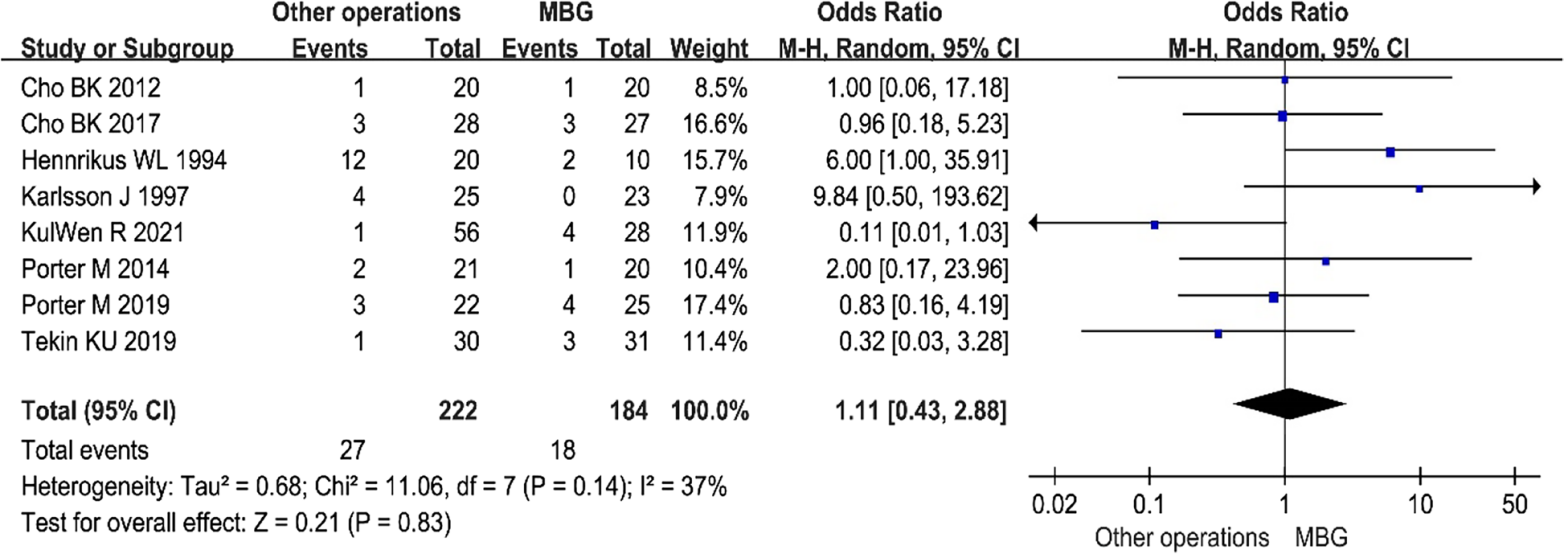
Complications

For the meta-analysis, four complications were chosen: infection, recurrence, irritation, and nerve injury. Figure 5 depicts the final result. We found no significant discrepancies and completed the meta-analysis using the fixed model to account for all problems. When assessing each complication, no statistical significance was identified between the two groups (MD = 1.11, 95% CI: 0.43– 2.88, *P* = 0.83).

## Discussion

Lateral ankle sprain (LAS) is a common disease for physically active people. It also has a high incidence in the general population. Conservative treatment procedures such as bracing and plaster fixing can heal about 80% of acute ankle sprains. CLAI continues to affect 20% of individuals [19]. The expert panel agreed on five critical factors in terms of injury diagnosis. The following are some of them: (1) previous lateral ankle sprain; (2) etiology of injury; (3) weight-bearing status; (4)clinical evaluation of ligaments, and (5) clinical evaluation of bones [20]; Delahunt et al. expanded the under-defined inclusion criteria in CLAI to classify people with mechanically and functionally unstable ankles. They clearly stated that: to be classified as CLAI, residual symptoms ("withdrawal" and a feeling of ankle instability) should be present at least one year after the initial sprain [21]. The diagnostic criteria are the difference between the lateral ankle joint and the opposite side when the talus is tilted 10° or more. The radiographic criteria for chronic lateral ankle instability surgery were > 15° or a side-to-side difference > 10° Of tibiotalar tilt angle on varus stress radiographs and > 10 mm or a side-to-side difference of > 3 mm of anteriortalar translation on anterior drawer stress radiographs [22]. Due to the high recurrence rate, patients, their families, and society need to bear a substantial medical burden [23].

With the improvement of living standards, the number of people with heavy weights has gradually increased. After sprained ankle joints, higher requirements for its stability have been put forward. Ligament repair and reconstruction, are two of CLAI's ligament repair treatments. MBG surgery refers to suturing the upper bundle of IER to the lower fibular segment based on the original operation; it is one of the most often utilized surgical procedures to treat CLAI. Other operations covered in this article, such as the LARS、AST、the procedure as reported by Karlsson et al. and the Chrisman-Snook procedure.

When the residual ligament of the patient is small, the use of MBG alone cannot provide satisfactory postoperative results, so other surgical methods can be considered to meet the needs of the patient. Regarding ligament reconstruction surgery, such as LARS, AST, etc. In the study of Hong Li et al., there are no substantial differences in risk of complications between Suture Tape Augmented repair and BR surgery; according to the researchers, the Suture Tape Augmented repair procedure seems to be a safe and fast option [24]. However, in one study, it was found that the MBG had less anterior talar displacement and talar tilting than the Chrisman-Snook or the incised ligament groups at all forces. Many scholars have recently compared other surgical methods with MBG to explore the best treatment method, Tekin Kerem Ulku et al. [14] found that the lateral ankle ligament suture augmentation is comparable to arthroscopic MBG, with a shorter operation and operation time. No casts or braces are required. William L. Henrik et al. [16] concluded that the MBG treated group had the lowest morbidity among the patients observed. The frequently mentioned complications of nerve entrapment and excessive lateral ankle tightening were less common. According to Stephen H. Liu et al. [18, 20], for ClAI with extensive ligamentous laxity, other procedures augmented with suture tape are a practical option. Some scholars believe that MBG provides excessive tension on the repaired ligament compared with other surgeries, resulting in more significant mechanical constraints than other techniques. As a method to improve the clinical outcome of CLAI. In the treatment of CLAI, various techniques have been matured. The author believes that different treatment methods can achieve sound therapeutic effects, and there is no significant difference in postoperative complications. Therefore, other surgical techniques also can be used for treatment, not only MBG.

There is currently no consistent scoring method for evaluating the prognosis of ankle stabilization surgery [25]. However, most studies used the American Orthopedic Foot and Ankle Association Ankle Posterior Foot Scale (AOFAS) score, stress radiographs, the Sefton Grading system, and patient satisfaction scores, these scoring methods are covered in relevant randomized trials [4, 5, 7, 10–14]. Using the scoring methods described above, we compared the results of the other operations groups and the MBG group. The other operations groups had significant advantages over the MBG group in all aspects of the FAOS score. The authors believe that the difference in FAOS scores is due to the use of tendons or suture tape to mimic the torn ligament's original physiological role and enhance ankle stability with more muscular tissue or material, thereby causing the difference in FAOS results. There was no significant difference between the two groups in the talar tilt angle and Anterior talar translation, both of which restored the average angle of the ankle joint. According to the results, other procedures can obtain better prognostic indicators than MBG and have a more comprehensive application range and shorter postoperative fixation time. According to the authors, other surgeries could be a good option for treating CLAI.

For postoperative ankle stability evaluation, radiographs were used to measure the talar tilt angle under a 150 N varus stress and the anterior talar translation under the anterior drawer stress. The Talar Tilt Angle is the angle created by the distal tibia's articular surfaces and the talus's articular surface while under varus stress, the method has been proven to be an accurate and practical method [26]. The mean talar tilt angle and anterior talar translation of patients in the two groups were over 10° and 10 mm before surgery, indicating lateral ligament damage in the ankle joint. In both groups of patients, the talar tilt angle and anterior talar translation dropped to the normal range after surgery, suggesting that CLAI can be stabilized. Since Other operations apply to an extensive range of people and have a shorter postoperative fixed period, they can obtain better prognostic indicators than MBG. The author believes that other operations can be a good choice for treating CLAI.

After pooling all the results, the other surgery group had an advantage in the FAOS score; there are no differences between the two groups in evaluating ankle stability and complications. Hence, the authors considered that the other operation group was better than the MBG group. In treating CLAI, other suitable surgical methods can be adopted, and MBG is not stubbornly used as the only choice.

## Study strengths and limitations

For postoperative ankle stability evaluation, radiographs were used to measure the talar tilt angle under a 150 N varus stress and the anterior talar translation under the anterior drawer stress. The Talar Tilt Angle is the angle created by the distal tibia's articular surface and the talus' articular surface under varus load. Before surgery, patients in both groups had a mean talar tilt angle of over 10° and anterior talar translation of over 10 mm, indicating lateral ligament injury in the ankle joint.

## Conclusion

The current meta-analysis found no significant differences between other operations and MBG operations regarding postoperative ankle stability evaluation, or complications. Based on the findings, the author feels that other operations surgery has clinical advantages over MBG surgery. While the data is favorable, due to the low methodological quality of RCTs, it is not conclusive. More high-quality RCTs with a low risk of bias and adequate sample numbers are needed to demonstrate its genuine effects.

## Data Availability

The datasets used and/or analyzed during the current study available from the corresponding author on reasonable request.

## Abbreviations

CLAI: Chronic Lateral Ankle Instability
MBG: The Modified Brostrom-Gould

## References

[1] Clanton TO, Campbell KJ, Wilson KJ, Michalski MP, Goldsmith MT, Wijdicks CA (2014). Qualitative and Quantitative Anatomic Investigation of the Lateral Ankle Ligaments for Surgical Reconstruction Procedures. J Bone Joint Surg Am.

[2] Viens NA, Wijdicks CA, Campbell KJ, Laprade RF, Clanton TO (2014). Anterior talofibular ligament ruptures, part 1: biomechanical comparison of augmented Brostrom repair techniques with the intact anterior talofibular ligament. Am J Sports Med.

[3] Matsui K, Burgesson B, Takao M, Stone J, Guillo S, Glazebrook M (2016). Minimally invasive surgical treatment for chronic ankle instability: a systematic review. Knee Surg Sports Traumatol Arthrosc.

[4] Porter M, Shadbolt B, Ye X, Stuart R. Ankle lateral ligament augmentation versus the modified brostrom-gould procedure: a 5-year randomized controlled trial. Am J Sports Med. 2019;47(3):659–66. 10.1177/0363546518820529. 10.1177/0363546518820529 30699039

[5] Hennrikus WL, Mapes RC, Lyons PM, Lapoint JM. Outcomes of the Chrisman-Snook and modified-Broström procedures for chronic lateral ankle instability. A prospective, randomized comparison. Am J Sports Med. 1996;24(4):400–4. 10.1177/036354659602400402. 10.1177/036354659602400402 8827297

[6] Karlsson J, Eriksson BI, Bergsten T, Rudholm O, Swärd L (1997). Comparison of two anatomic reconstructions for chronic lateral instability of the ankle joint. Am J Sports Med..

[7] Porter M, Shadbolt B, Stuart R. Primary ankle ligament augmentation versus modified Brostrom-Gould procedure: a 2-year randomized controlled trial. ANZ J Surg. 2015;85(1–2):44–8. 10.1111/ans.12837. 10.1111/ans.12837 25171115

[8] Moher D, Liberati A, Tetzlaff J, Altman DG (2009). Preferred reporting items for systematic reviews and meta-analyses: the PRISMA statement. PLoS Med.

[9] Higgins JP, Altman DG, Gotzsche PC, Juni P, Moher D, Oxman AD (2011). The Cochrane Collaboration's tool for assessing risk of bias in randomised trials. BMJ.

[10] Cho BK, Park JK, Choi SM, SooHoo NF (2019). A randomized comparison between lateral ligaments augmentation using suture-tape and modified Brostrom repair in young female patients with chronic ankle instability. Foot Ankle Surg.

[11] Karlsson J, Eriksson BI, Bergsten T, Rudholm O, Swärd L (1997). Comparison of two anatomic reconstructions for chronic lateral instability of the ankle joint. Am J Sports Med.

[12] Cho BK, Kim YM, Kim DS, Choi ES, Shon HC, Park KJ. Comparison between suture anchor and transosseous suture for the modified-Brostrom procedure. Foot Ankle Int. 2012;33(6):462–8. 10.3113/FAI.2012.0462. 10.3113/FAI.2012.0462 22735317

[13] Kulwin R, Watson TS, Rigby R, Coetzee JC, Vora A. Traditional Modified Brostrom vs Suture Tape Ligament Augmentation. Foot Ankle Int. 2021;42(5):554–61. 10.1177/1071100720976071. 10.1177/1071100720976071 33491480

[14] Tekin KU. Arthroscopic suture-tape internal bracing is safe as arthroscopic modified Broström repair in the treatment of chronic ankle instability. 10.1007/s00167-019-05552-w 31197389

[15] Ulku TK, Kocaoglu B, Tok O, Irgit K, Nalbantoglu U. Arthroscopic suture-tape internal bracing is safe as arthroscopic modified Broström repair in the treatment of chronic ankle instability. Knee Surg Sports Traumatol Arthrosc. 2020;28(1):227–32. 10.1007/s00167-019-05552-w. Epub 2019 Jun 13. 10.1007/s00167-019-05552-w 31197389

[16] Cho BK, Park KJ, Park JK, SooHoo NF. Outcomes of the modified broström procedure augmented with suture-tape for ankle instability in patients with generalized ligamentous laxity. Foot Ankle Int. 2017;38(4):405–11. 10.1177/1071100716683348. 10.1177/1071100716683348 28367693

[17] Halasi T, Kynsburg A, Tállay A, Berkes I (2005). Changes in joint position sense after surgically treated chronic lateral ankle instability. Br J Sports Med.

[18] Delahunt E, Bleakley CM, Bossard DS, Caulfield BM, Docherty CL, Doherty C, Fourchet F, Fong DT, Hertel J, Hiller CE, Kaminski TW, McKeon PO, Refshauge KM, Remus A, Verhagen E, Vicenzino BT, Wikstrom EA, Gribble PA. Clinical assessment of acute lateral ankle sprain injuries (ROAST): 2019 consensus statement and recommendations of the International Ankle Consortium. Br J Sports Med. 2018;52(20):1304–10. 10.1136/bjsports-2017-098885. Epub 2018 Jun 9. 10.1136/bjsports-2017-098885 29886432

[19] Hu CY, Lee KB, Song EK, Kim MS, Park KS (2013). Comparison of bone tunnel and suture anchor techniques in the modified Brostrom procedure for chronic lateral ankle instability. Am J Sports Med.

[20] Li HY, Guo A, Yang F, Zheng JJ, Hua YH, Chen SY (2021). The anterior talofibular ligament-posterior talofibular ligament angle decreased after ankle lateral stabilization surgery. Knee Surg Sports Traumatol Arthrosc.

[21] Vuurberg G, Pereira H, Blankevoort L, van Dijk CN (2017). Anatomic stabilization techniques provide superior results in terms of functional outcome in patients suffering from chronic ankle instability compared to non-anatomic techniques. Knee Surg Sports Traumatol Arthrosc.

[22] Choi JH, Choi KJ, Chung CY, Park MS, Sung KH, Lee KM (2021). Consistency and Reliability of Ankle Stress Radiography in Patients With Chronic Lateral Ankle Instability. Orthop J Sports Med.

[23] Gribble PA, Bleakley CM, Caulfield BM, Docherty CL, Fourchet F, Fong DT (2016). Evidence review for the 2016 International Ankle Consortium consensus statement on the prevalence, impact and long-term consequences of lateral ankle sprains. Br J Sports Med.

[24] Li H, Zhao Y, Chen W, Li H, Hua Y (2020). No Differences in Clinical Outcomes of Suture Tape Augmented Repair Versus Broström Repair Surgery for Chronic Lateral Ankle Instability. Orthop J Sports Med.

[25] Cho BK, Park KJ, Kim SW, Lee HJ, Choi SM (2015). Minimal Invasive Suture-Tape Augmentation for Chronic Ankle Instability. Foot Ankle Int.

[26] Beynnon BD, Webb G, Huber BM, Pappas CN, Renstrom P, Haugh LD (2005). Radiographic measurement of anterior talar translation in the ankle: determination of the most reliable method. Clin Biomech (Bristol, Avon).

